# Supplementary material for: Identification of a Musashi2 translocation as a novel oncogene in myeloid leukemia
Source: bioRxiv. 2023 Dec 29:2023.12.29.573601. Preprint. [Version 1] doi: 10.1101/2023.12.29.573601 (PMC10793452; doi:10.1101/2023.12.29.573601)
Supplement: Supplement 1 [file NIHPP2023.12.29.573601V1-supplement-1.pdf]

bioRxiv preprint doi: https://doi.org/10.1101/2023.12.29.573601; this version posted December 29, 2023. The copyright holder for this preprint (which was not certified by peer review) is the author/funder, who has granted bioRxiv a license to display the preprint in perpetuity. It is made available under aCC-BY 4.0 International license.

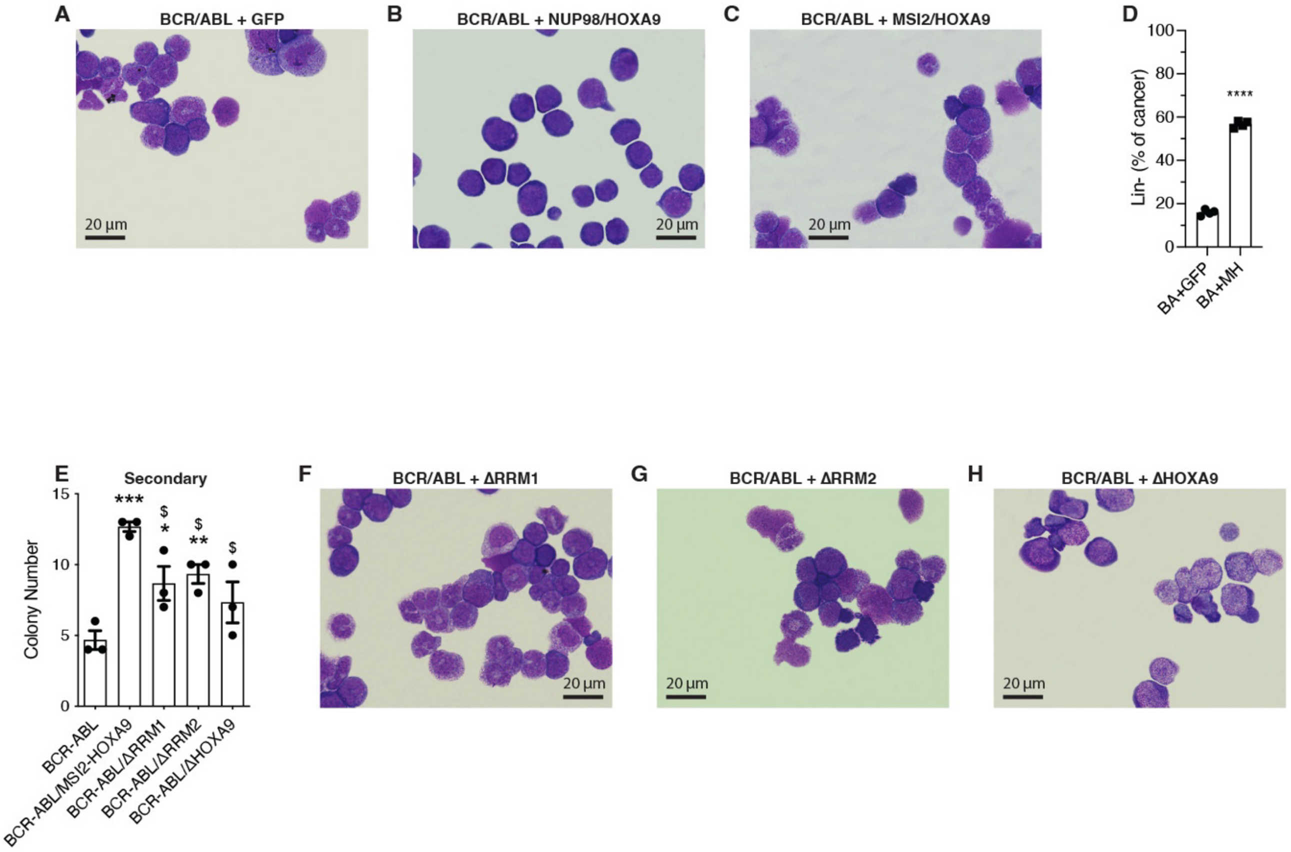

Figure S1. Analysis of BCR-ABL/MSI2-HOXA9 leukemia

(*A-C*) Representative brightfield image of (*A*) BCR-ABL/Control, (*B*) BCR-ABL/NUP98-HOXA9, (*C*) BCR-ABL/MSI2-HOXA9 cytospins of lin- cancer stained with Giemsa and May-Grunwald stains. (*D*) Quantification of BCR-ABL/MSI2-HOXA9 and BCR-ABL Lin- content within the cancer population by FACS. ****P<0.0001, n=4 for each group. (*E*) Secondary colony formation of KLS cells expressing BCR-ABL/Control or BCR-ABL + variations of the MSI2-HOXA9 fusion protein. *=significance from BCR-ABL/Control, *P=0.04, **P=0.007 ***P=0.0004; $=significance from BCR-ABL/MSI2-HOXA9, $P=0.03 for ΔRRM1, $P=0.01 for ΔRRM2, $P=0.02 ΔHOXA9 (n=3 technical replicates). (*F-H*) Representative brightfield image of (*F*) BCR-ABL/ΔRRM1, (*G*) BCR-ABL/ΔRRM2 (*H*) BCR-ABL/ΔHOXA9 cytospins of lin- cancer stained with Giemsa and May-Grunwald stains.

bioRxiv preprint doi: https://doi.org/10.1101/2023.12.29.573601; this version posted December 29, 2023. The copyright holder for this preprint (which was not certified by peer review) is the author/funder, who has granted bioRxiv a license to display the preprint in perpetuity. It is made available under aCC-BY 4.0 International license.

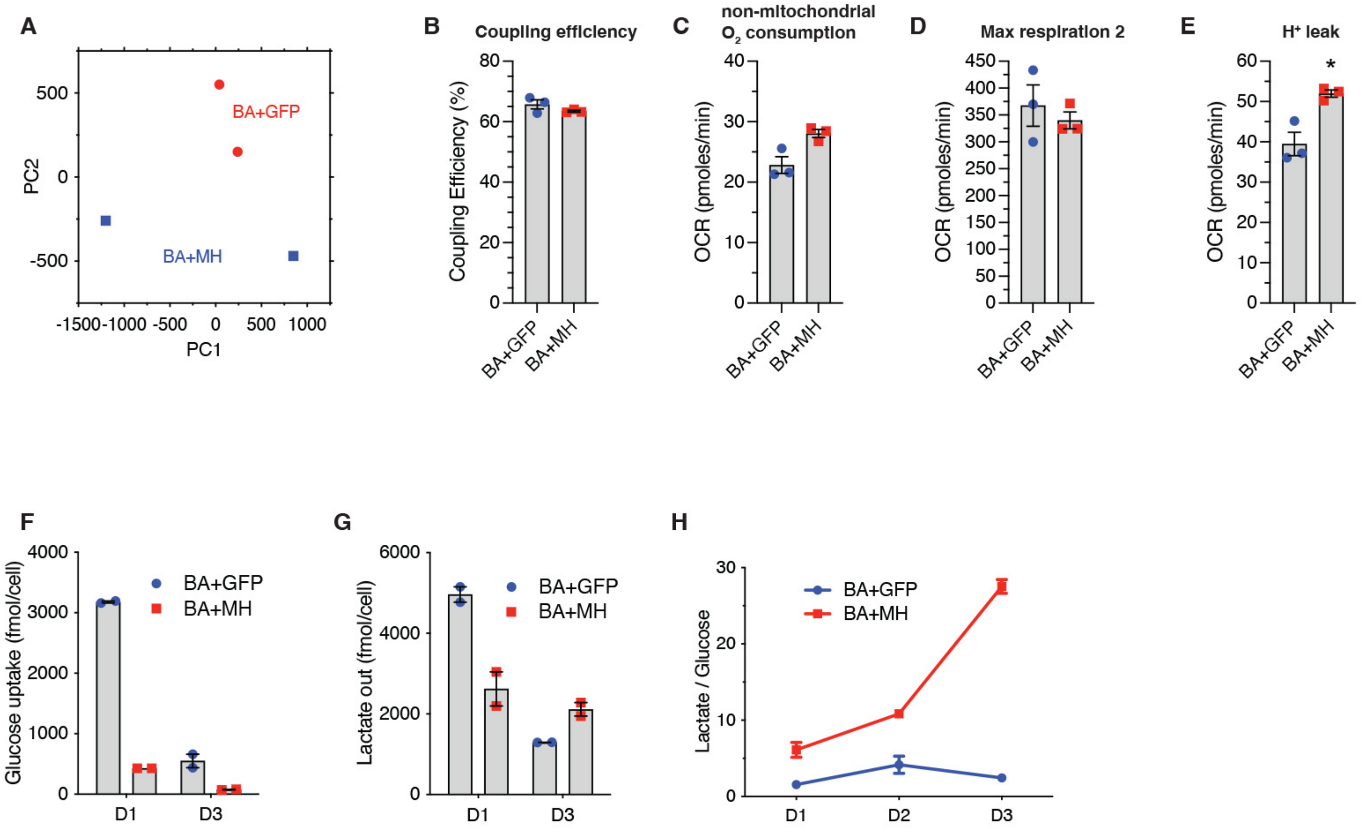

Figure S2. Principal component analysis and mitochondrial parameters associated with BCR-ABL/MSI2-HOXA9 leukemia

(*A*) Principal component analysis of RNAseq data of Lin- BCR-ABL/Control and Lin- BCR-ABL/MSI2-HOXA9. (*B*) Coupling efficiency determined from OCR of Lin- BCR-ABL/Control and Lin- BCR-ABL/MSI2-HOXA9 (n=3 technical replicates). (*C*) Non-mitochondrial O_2 consumption determined from OCR of Lin- BCR-ABL/Control and Lin- BCR-ABL/MSI2-HOXA9 (n=3 technical replicates). (*D*) Maximum respiration determined from OCR of Lin- BCR-ABL/Control and Lin- BCR-ABL/MSI2-HOXA9 following the second FCCP injection, an oversaturating concentration (n=3 technical replicates). (*E*) Proton leak determined from OCR of Lin- BCR-ABL/Control and Lin- BCR-ABL/MSI2-HOXA9. *P=0.01 (n=3 technical replicates). (*F*) Glucose uptake of Lin- BCR-ABL/Control and Lin- BCR-ABL/MSI2-HOXA9 measured by the YSI bioanalyzer at day 1 (D1) and day 3 (D3) post-plating (n=2 technical replicates). (*G*) Lactate production of Lin- BCR-ABL/Control and Lin- BCR-ABL/MSI2-HOXA9 measured by the YSI bioanalyzer at day 1 (D1) and day 3 (D3) post-plating (n=2 technical replicates). (*H*) Ratio of lactate production to glucose consumption of Lin- BCR-ABL/Control and Lin-BCR-ABL/MSI2-HOXA9 at day 1 (D1), day 2 (D2), and day 3 (D3) post-plating (n=2 technical replicates).

bioRxiv preprint doi: https://doi.org/10.1101/2023.12.29.573601; this version posted December 29, 2023. The copyright holder for this preprint (which was not certified by peer review) is the author/funder, who has granted bioRxiv a license to display the preprint in perpetuity. It is made available under aCC-BY 4.0 International license.

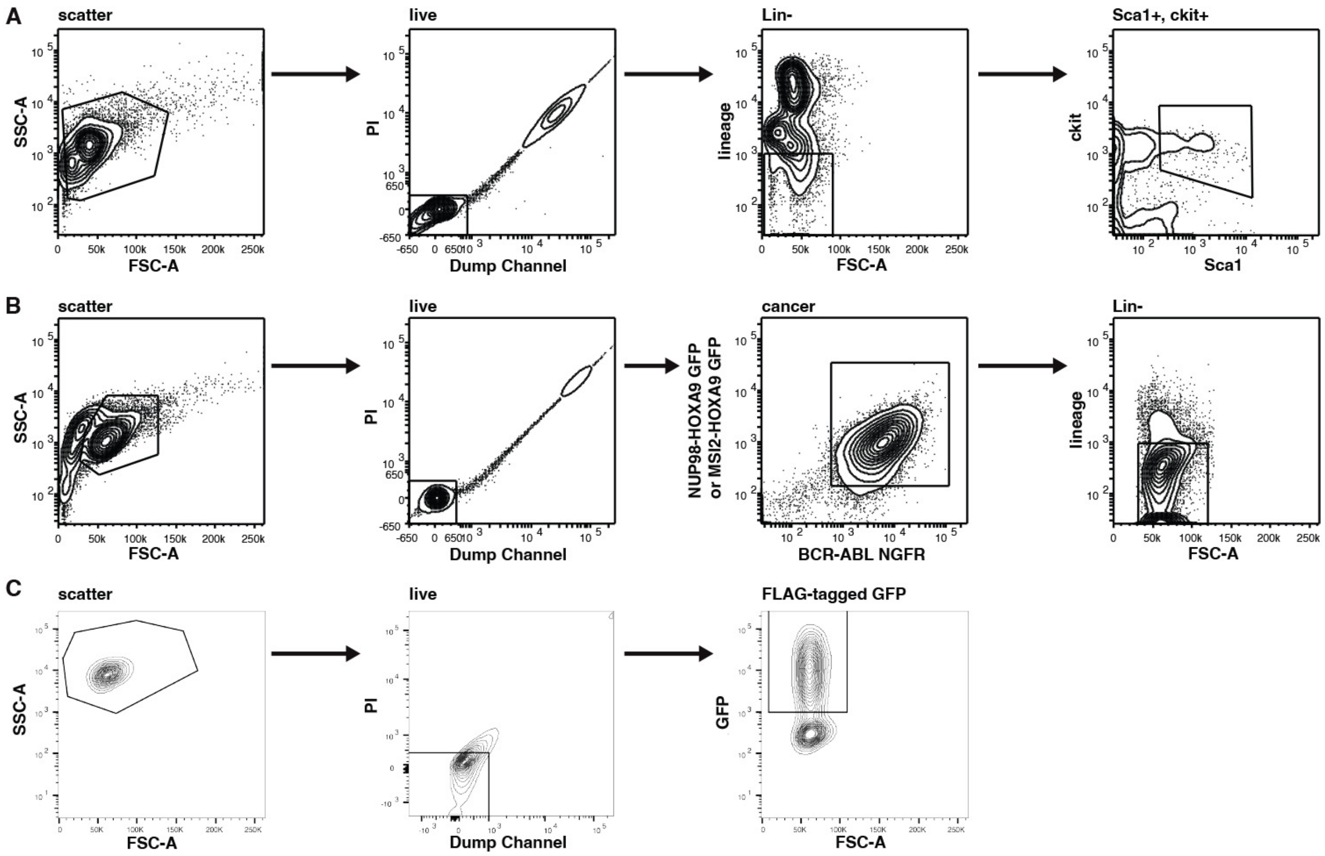

Figure S3. Examples of flow cytometry gating strategies

(*A*) Gating strategy used for KLS sorting from mouse bone marrow. KLS cells were used as starting material for all mouse leukemia models in this paper. (*B*) Gating strategy used for sorting lin- bcCML from primary transplants. This strategy was used to generate the cell source for histology, transplants, and RNAseq. (*C*) Gating strategy used for isolating FLAG-tagged transduced K562 cells. After 48hrs in the presence of BCR-ABL + FLAG-tagged wild-type MSI2 or FLAG-tagged MSI2/HOXA9, cells were sorted to isolate doubly infected cells. This strategy was used for the localization experiments (Fig. 5B-D).
